# Diagenetic evolution of the Oligocene Huagang Formation in Xihu sag, the East China Sea Shelf Basin

**DOI:** 10.1038/s41598-020-76481-9

**Published:** 2020-11-10

**Authors:** Wendao Qian, Taiju Yin, Changmin Zhang, Huijia Tang, Guowei Hou

**Affiliations:** 1 School of Geosciences, Yangtze University, Wuhan, 430100 China; 2 School of Earth Sciences, China University of Geosciences, Wuhan, 430074 China; 3 Shanghai Branch, CNOOC Ltd., Shanghai, 200030 China

**Keywords:** Geodynamics, Geology, Mineralogy, Sedimentology

## Abstract

The deep and ultra deep clastic reservoir is characterized by strong reservoir heterogeneity and complicated reservoir-forming characteristics for its high degree of diagenetic stage and the complexity of diagenesis. In order to better study the diagenetic evolution of deep and ultra deep reservoir in the burial process, a fine subsection scheme of 36 diagenetic micro-stage (DS) in diageneitic process was proposed based on paleotemperature (T), vitrinite reflectance (*Ro*%) and proportion of smectite in illite/smectite interstratified minerals (I/S-S%). Taking the Oligocene Huagang Formation in the Xihu sag of the East China Sea Basin as an example, the diagentic stage IIA1–IIA2–IIB was identified mainly by means of formation temperature data (T), homogenization temperature of fluid inclusion (Th), vitrinite analysis and clay mineral X-ray diffraction method. On this basis, diagenetic evolution using the fine subsection scheme in geological time were conducted. This fine division of diagenetic stage could bring accurate insight into porosity evolution history, hydrocarbon charging periods, diagenetic and reservoir-forming characteristics of low-permeability and tight sandstone.

## Introduction

With the development of petroleum exploration and exploitation, deep (3500–4500 m) to ultra-deep (> 4500 m) tight sandstone has been paid more attention because of its abundant oil and gas resources^1,2^. The deep and ultra deep clastic reservoir is characterized by strong reservoir heterogeneity and complicated reservoir-forming characteristics for its high degree of diagenetic stage and the complexity of diagenesis^3–5^. Considerable efforts have been made to understand the spatial and temporal distribution of diagenesis in clastic rocks, maily due to the realization that these alterations exert profound controls on reservoir quality^6–9^. It is now evident that fluid properties, diagenesis of clastic rock and hydrocarbon generating capacity are closely related with diagenetic stages, and thus research on diagenetic evolution has important significance for exploration and exploitation of low-permeability and tight sandstone^3,8,10–13^.

The recognization that diagenetic evolution has the characteristics of stages^14–17^ has already begun in the early 1970s, and Schmidt and Morad (1979)^18^ first put forward the division basis and naming method of diagenetic stages. Domestic scholars have also proposed the division standard of diagenetic stages of clastic rocks on basis of previous research^11,19,20^ (Table 1). In recent years, a subdividing the intermediate diagenetic stage A into four diagenetic microfacies was proposed using vitrinite reflectance^17^. Throughout those researches, the division theory of diagenetic stage from qualitative and quantitative perspectives is mainly based on the diagenetic environment parameters such as temperature, time, pressure, fluid, while the contact relationship of clastic particles, pore structure, authigenic mineral assemblage, vitrinite reflectance (*Ro*%), peak temperature of organic matter solution (Tmax), clay mineral conversion rate (I/S-S%), steranes horane isomerization index (SI) and quartz secondary enlargement index are integrated.

**Table 1:** Division scheme of diagenetic stage of clastic reservoir by Ying (2003).

Diagenetic stages		Paleo temperature (℃)	Organic matter	Mudstone
D	Ying’s symbol		*Ro*%	I/S-S%
D1	IA	≤ 65	< 0.35	> 70
D2	IB	> 65–85	0.35–0.55	70–50
D3	IIA1	> 85–110	0.55–0.7	50–20
D4	IIA2	> 110–140	0.7–1.3	20–15
D5	IIB	> 140–175	1.3–2.0	< 15
D6	III	> 175	> 2.0	≈0

IA—early diagenetic stage A; IB—early diagenetic stage B; IIA1—middle diagenetic stage A1; IIA2—middle diagenetic stage A2; IIB—middle diagenetic stage B; III—late diagenetic stage.

The Oligocene Huagang Formation (E_3_h) with buried depth from 2000 to 6000 m is a vital gas producing formation in the Xihu sag, East China Sea Basin^22–24^. In the past five decades, lots of oil and gas fields and structures have been discovered in the exploration of Xihu sag of the East China Sea Basin. For the sandstone reservoir of the E_3_h Formation, previous scholars mainly focused on the following aspects: (1) lithologic features, diagenetic environment evolution and controlling factors of the Oligocene Huagang formation tight sandstone on the central inverted structural belt^25–28^; (2) the tectonic activities on diagenetic and reservoir-forming process in the Upper section of Huagang formation, including how tectonic activities controlled the differential burial process and hydrocarbon generation history of source rock , and how fracture systems connect source rocks with reservoirs on the central inverted structural belt^22,29–31^; and (3) sedimentary source and sedimentary environment changing through space and time^16,32,33^. However, the limited offshore geologic data restricted offshore oil and gas exploration in the deep formation. Reservoir diagenetic evolution and reservoir forming evolution characteristics has important guiding significance for improving exploration success rate and expanding exploration depth. Therefore, in order to better study the diagenetic evolution of deep and ultra deep reservoir in the burial process, the reservoir lithologies, diagenesis types, pores structure, fluid inclusions on quartz transgranular crack and quartz overgrowth, characteristics of organic matter, burial history and thermal history were conducted by means of thin section, scanning electron microscope, homogenization temperature of fluid inclusion, bulk rock X-ray diffraction analysis, cathodoluminescence (CL), clay mineral X-ray diffraction analysis, and a fine subsection scheme of 36 diagenetic micro-stage (DS) in diageneitic process was proposed based on paleotemperature (T), vitrinite reflectance (*Ro*%) and proportion of smectite in illite/smectite interstratified minerals (I/S-S%).The purposes of this fine division of diagenetic stage are to: (1) improve the understanding of low-permeability and tight sandstone reservoirs diagenetic evolution; (2) discuss the difference of T, *Ro*% and I/S-S% in the division of diagenetic stages; (3) reveal the geological factors affecting the differential evolution; (4) bring accurate insight into porosity evolution history, hydrocarbon charging periods, diagenetic and reservoir-forming characteristics of low-permeability and tight sandstone.

## Geological setting

**Figure 1:**
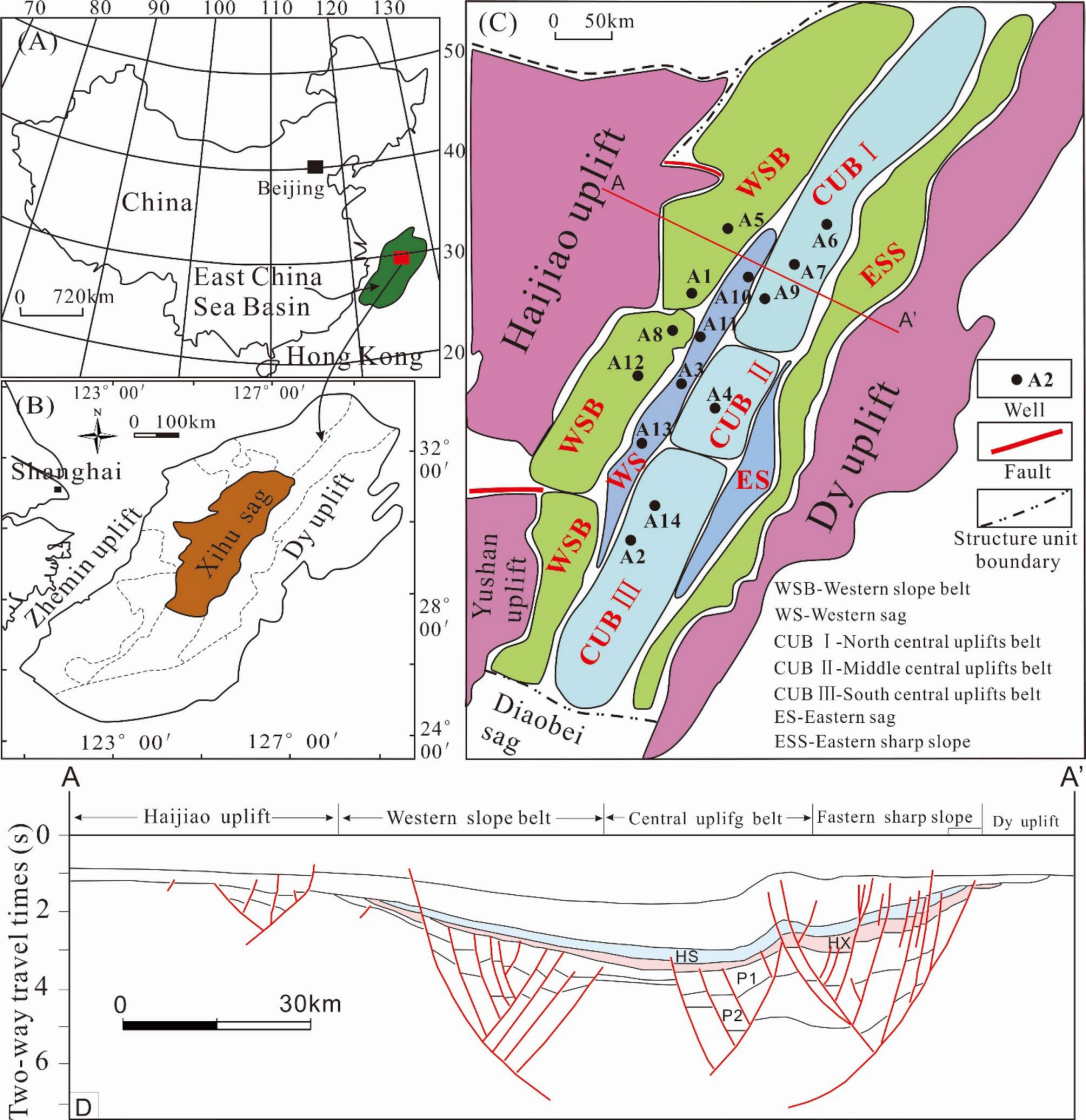
Structural map of Xihu sag in East China Sea Shelf Basin (modified from Wang et al., 2020). **(A)** Geographical location of the East China Sea Basin; **(B)** location map of the Xihu sag, East China Sea Basin; **(C)** tectonic units of the Xihu sag (by CorelDRAW2016, https://www.corel.com/cn/); **(D)** profile of AA’ across the Xihu sag.

**Figure 2:**
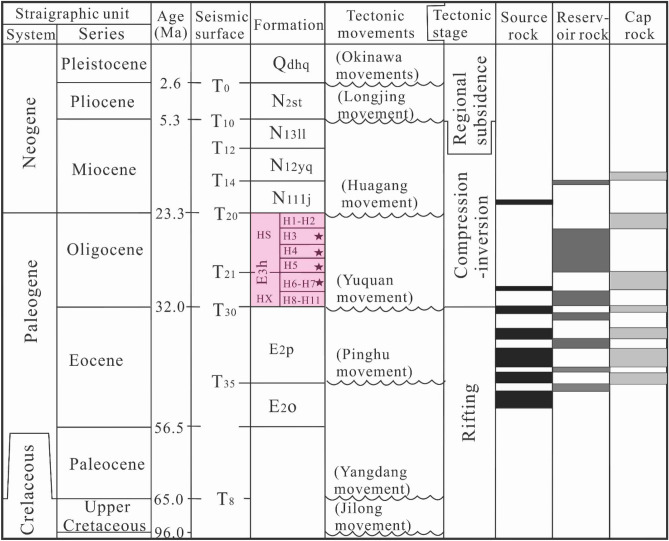
Cenozoic-Quaternary stratigraphy column of the Xihu sag (modified from Wang et al., 2020). The Oligocene H3, H4, H5, H6 and H7 members were highlighted by black stars.

The Xihu sag with about 440 km long from north to south, 110 km wide from east to west and an area of 51,800 km^2^ is situated in the East China Sea Shelf Basin and generally oriented in NNE direction^23,33,34^ (Fig. 1). In the light of evolutionary characteristics of deposition and structures in the course of its formation and development, the Xihu sag can be divided into five structural zones as follows: Western slope belt (WSB), Western sag (WS), Central uplift belt (CUB), Eastern sag (ES) and Eastern sharp slope (ESS)^23,33,34^ (Fig. 1C). The formation and evolution of the Xihusag have experienced five major tectonic movements, called Keelung movement (TG), Pinghu movement (T40), Yuquan movement (T30), Huagang movement (T20), Longjing movement (T12) and Okinawa Trough movement respectively, which formed six regional unconformities in the sag. Among them, Yuquan movement (T30) and Huagang movement (T20) divided the structural layer of Xihu sag into three parts from bottom to top: rift structural layer Synrift stage (Tg–T30), sag structural layer (T30–T12) and regional subsidence structural layer (T12-seafloor), and Yuquan movement at the end of Eocene and Longjing movement at the end of Miocene are two important periods of structural change in Xihu sag. The regional structural evolution of Xihu sag can be roughly divided into three stages: synrift stage, depression stage, regional subsidence stage^23,24^. The Cretaceous Shimentan formation, Eocene Bajiaoting formation, Baoshi formation, Pinghu Formation, Oligocene Huagang Formation, Miocene Longjing formation, Yuquan formation and Liulang formation, Pliocene Santan formation and quaternary Donghai group are developed in Xihu sag from the bottom to the top, while the Paleocene stratum is not developed in the sag^24,35^(Fig. 2). The main target reservoir in the Xihu sag is the Eocene Pinghu formation and the Oligocene Huagang formation. The Huagang formation can be subdivided into the upper and lower members (HS and HX)^22,29^. The main stratigraphic features are as follows. (1) The lower member of the Huagang formation was deposited in the early stage of depression, but it was closed by retrogression in the late stage of the Pinghu movement and transitioned to lacustrine deposits. The thickness revealed by drilling was 80–829 m. The lithology mainly consists of a thin layer of glutenite, gravel sandstone, coarse sandstone, medium sandstone, fine sandstone, siltstone, argillaceous siltstone, mudstone, and the local entrapment of a thin coal seam, reflecting a sedimentary period with adequate sediment sources. (2) The upper member of the Huagang formation was deposited in the early stage of the depression. A lacustrine transgression and retrogression occurred again after the lacustrine regression. The lacustrine transgression was large in scale, and the thickness revealed by drilling was 105–1100 m. The lithology is coarse and mainly consists of a thin layer of glutenite, gravelly sandstone, coarse sandstone, medium sandstone, fine sandstone, siltstone, argillaceous siltstone, mudstone, shale, and oil shale, reflecting a sedimentary period with adequate sediment sources.

## Database and methods

This study focused on the Oligocene H3, H4, H5, H6 and H7 sandstones and mudstones from fourteen wells in the Xihu sag (Fig. 1C). 43 fluid inclusion temperature test, 220 vitrinite reflectance test, 31 Rock–Eval test were conducted in the lab while 19 clay X-ray diffraction analysis were collected from the oilfield. The formation temperature from 22 samples was based on oil testing results.

### Burial history and thermal history analysis

The burial history and thermal history of the 14 wells were carried out by petrolmod2013 software. The depth, thickness, lithology and other data needed for the simulation process are mainly from the drilling and logging data, and the data of geological age, formation denudation, and heat flow value of the study area are from previous research results^22,36^.

### Fluid inclusion temperature test

43 core samples were prepared as thick doubly polished thin sections for fluid inclusion petrographic analyses and microthermometric measurements. The analyses were completed at the Reservoir Geology Experiment of Yangtze University by T600 cold and hot platform. DM4500P polarizing/fluorescence microscope (Leica, 25-1000) and Axio Imager. A2m polarizing/fluorescence microscope (Zeiss, 50–500 ×) were used for inclusion-petrography analysis. Inclusions on quartz transgranular crack and quartz overgrowth were tested. During the test, the inclusions were first cooled to – 70 ℃. During the heating process, when the temperature was between – 70 and − 20℃ and 0–70℃, the heating speed of the cooling and heating table was 20 ℃/min, and when the temperature was – 20 to 0 ℃ and 70–180 ℃, the heating speed of the cooling and heating table was 5 ℃/min. At room temperature, most of the salt water inclusions tested are two-phase fluid inclusions, and the test error is ± 1 ℃. Temperatures were measured and recorded when inclusions were completely homogeneous and completely dissolved.

### Vitrinite reflectance (*Ro*%) test

*Ro*% test was carried out with German Zeiss Axio Scope. A1/J&M MSP 200 and USA 3-Y international Limited DETA V4000-SP microscope spectrophotometer at the Reservoir Geology Experiment of Yangtze University. The measured light sheet was first placed on the platform of the microphotometer, and then moved with a mechanical moving ruler for measurement when it was in focus. During the measurement, quality control over the whole process were presented to ensure that there were no polishing defects in the measurement area and no interference of high reflectivity materials such as pyrite. When *Ro*% ≤ 0.5%, the number of measurement points is 25, and when *Ro*% > 0.5%, the number of measurement points is 30.

### Tmax test

The OGE-VI oil and gas evaluation workstation of CNPC at the Reservoir Geology Experiment of Yangtze University was used for rock pyrolysis analysis. The samples were heated in helium flow. The free gaseous hydrocarbon, free liquid hydrocarbon and pyrolytic hydrocarbon were detected by hydrogen flame ionization detector. The carbon dioxide discharged from pyrolysis and generated by the heated oxidation of pyrolytic residual organic matter were detected by thermal conductivity detector. Pyrolysis peak temperature (Tmax) is the pyrolysis temperature corresponding to the highest point of S2.

### The division of diagenetic stage

Based on the burial history and thermal history, with the help of Sweeney’s chemical kinetic method^37^, *Ro*% at different historical times can be calculated. The transformation process from smectite to illite is mainly affected by temperature, time, pressure, sedimentary environment and source characteristics^21,38,39^, which can be expressed by Elliot’s model formula^40^. In this study, the influence of four geological factors, temperature, pressure, fluid and time on diagenesis was considered comprehensively, while three parameters including palegeotemperature T (℃), vitrinite reflectance (*Ro*%) and smectite content in mixed layer (I/S-S%) were selected as the main parameters of comprehensive diagenetic evolution simulation to simulate the distribution of time domain and space domain and to divide the diagenetic stage in geological time.

## Results

### Formation temperature

**Figure 3:**
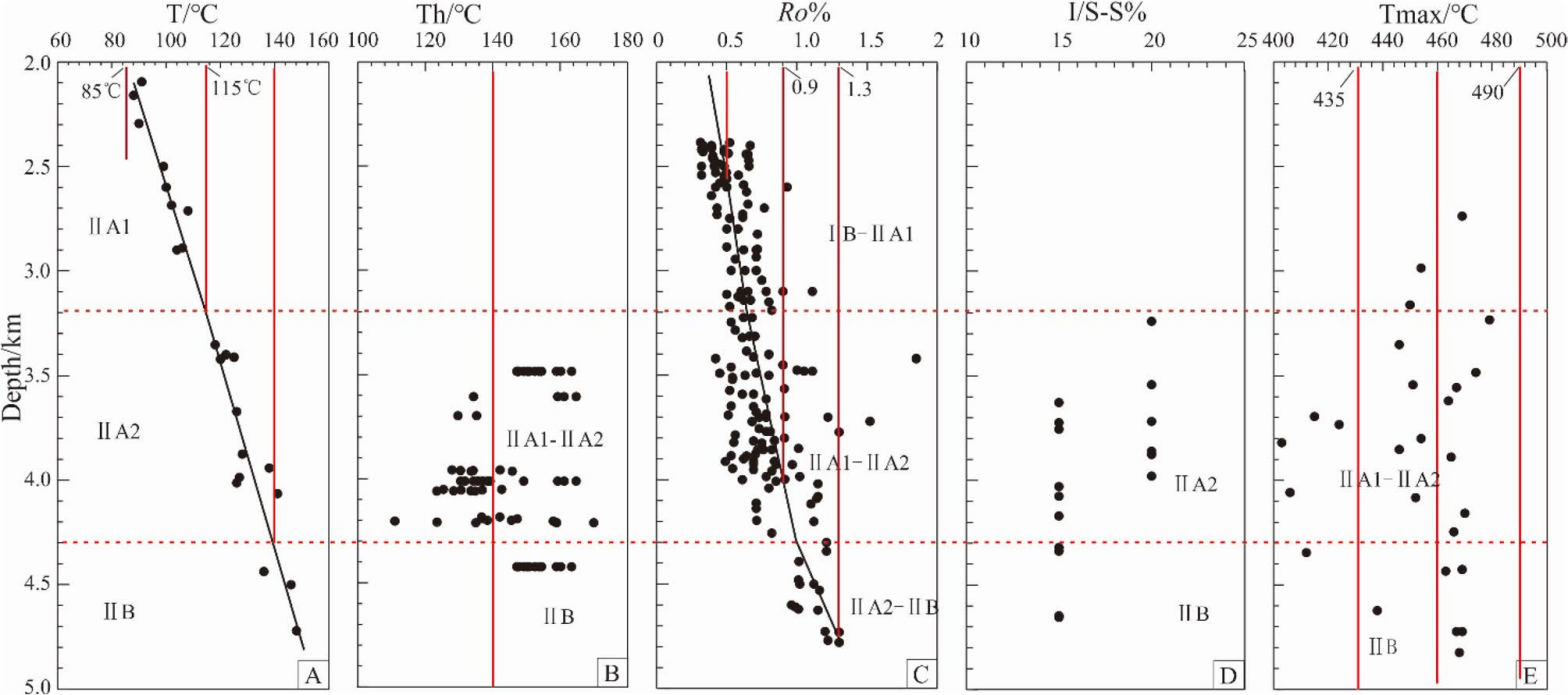
Vertical variation of T, Th, *Ro*%, I/S-S%, Tmax in Xihu sag and diagenetic stage division. **(A)** Formation temperature; **(B)** homogenization temperature of inclusions; **(C)** vitrinite reflectance; **(D)** smectite content in the I/S mixed layer; **(E)** pyrolysis peak temperature.

From the shallow layer to the deep layer, the current average geothermal gradient of Xihu sag is 30.5 ℃/km, and the current formation temperature is about 167 ℃ at 5000 m buried depth. The relationship between formation temperature and depth (Fig. 3) (Table 2) can be fitted as T = 0.0305*H + 15, R^2^ = 0.8726, in which T is formation temperature (℃), H is the buried depth (m), and the constant 15 is the average surface temperature. The present geothermal gradient in Xihu sag is higher in the Central inversion structural belt with 33.4 ℃/km and lower in the Western slope belt with 27.8 ℃/km and the Western sub sag with 30.5 ℃/km, in general lower than 3–5.5 ℃/100 m (Fig. 3A).

**Table 2:** Basic information of test samples from Huagang formantion in Xihu sag and corresponding analysis data.

Well	Layer	Depth (m)	T (℃)	Th (℃)	*Ro*%	I/S-S%	Tmax (℃)
A3	HS	2662–3551	(101–121)/115.0		(0.31–1.11)/0.59	20	–
A3	HX	3551–3880	(122–138)/130.0	(127.9–147.3)/137.6	(0.68–1.01)/0.79	(15–20)/12.5	–
A4	HS	2782–3733	–	–	(0.63–0.91)/0.74	–	(424–454)/445.2
A4	HX	3733–4068	–	–	(0.69–1.02)/0.85	–	–
A5	HX	3245–3458.5	–	(123.4–142.7)/132.2	(0.53–1.05)/0.83	–	–
A6	HS	2968–4225	–	(111–170)/144.0	–	–	(403–467)/433.5
A6	HX	4225–4460	–	–	–	–	–
A7	HS	3082–4426.5	–	–	–	(15–20)/16.4	–
A7	HX	4426.5–4800	–	–	–	15	(464–470)/467
A9	HS	2474–3634	(80–108)/87.9	(147.2–163.4)/152.9	(0.42–1.08)/0.78	–	–
A9	HX	3634–4333	–	(130.5–164.8)/143.8	–	–	(415–479)/458.2
A10	HS	3672–4661	–	(134.3–164.6)/152.7	–	–	(438–469)/461.8
A11	HX	3797–4419	(126–146)/133.3	–	–	(15–20)/16.7	–
A12	HS	2140–2576	–	–	(0.31–0.51)/0.39	–	–
A12	HX	2576–2850	–	–	(0.39–0.72)/0.51	–	–
A13	HS	2388.5–3172.5	(97–113)/109	–	(0.45–0.90)/0.65	–	–
A13	HX	3172.5–3760	(122–138)/130.0	–	(0.91–1.30)/1.11	–	–
A14	HS	2090–2927.5	(91–106)/99.6	–	–	–	–
A14	HX	2927.5–3401	(118–125)/121.5	–	–	–	–

### X-ray diffraction (XRD) of clay minerals

It was showed that the I/S-S% of Oligocene Huagang Formation in Xihu sag obtained by X-ray diffraction ranges from 15 to 20% (Fig. 3D) (Table 2), indicating that I/S layer has partially transformed into illite, and the source rock has reached a high maturity stage.

### Vitrinite reflectance *Ro*%

**Figure 4:**
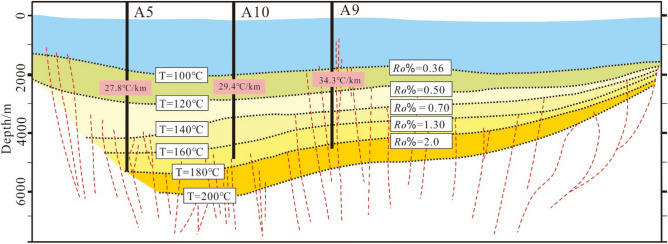
Vertical variation of T and *Ro*% in different oil fields.

According to the measured reflectance of vitrinite formation (*Ro*%) in Xihu sag (Fig. 3C) (Table 2), the value of *Ro*% has a significant increase trend with the burial depth. However, the value of *Ro*% is only about 1.3% when the burial depth of the stratum reaches 4500 m, and while the burial depth of the stratum is more than 5000 m, most of the value of *Ro*% is not more than 2%. This result showed that the rate of diagenetic evolution of Xihu sag is slow, while the degree of organic matter evolution is only in the middle diagenetic stage, and the maturity of organic matter is mostly in the low maturity maturity stage. In addition, through the analysis of the diagenetic evolution stages of different gas fields in the Western slope belt (A5), the Western sag (A10) and the Central uplift belt (A9) (Fig. 4), it is believed that the stratum is still in stage IIA at a burial depth of less than 4000 m and only start to evolve to stage IIB below 4000 m.

### Homogenization temperature of inclusions

**Figure 5:**
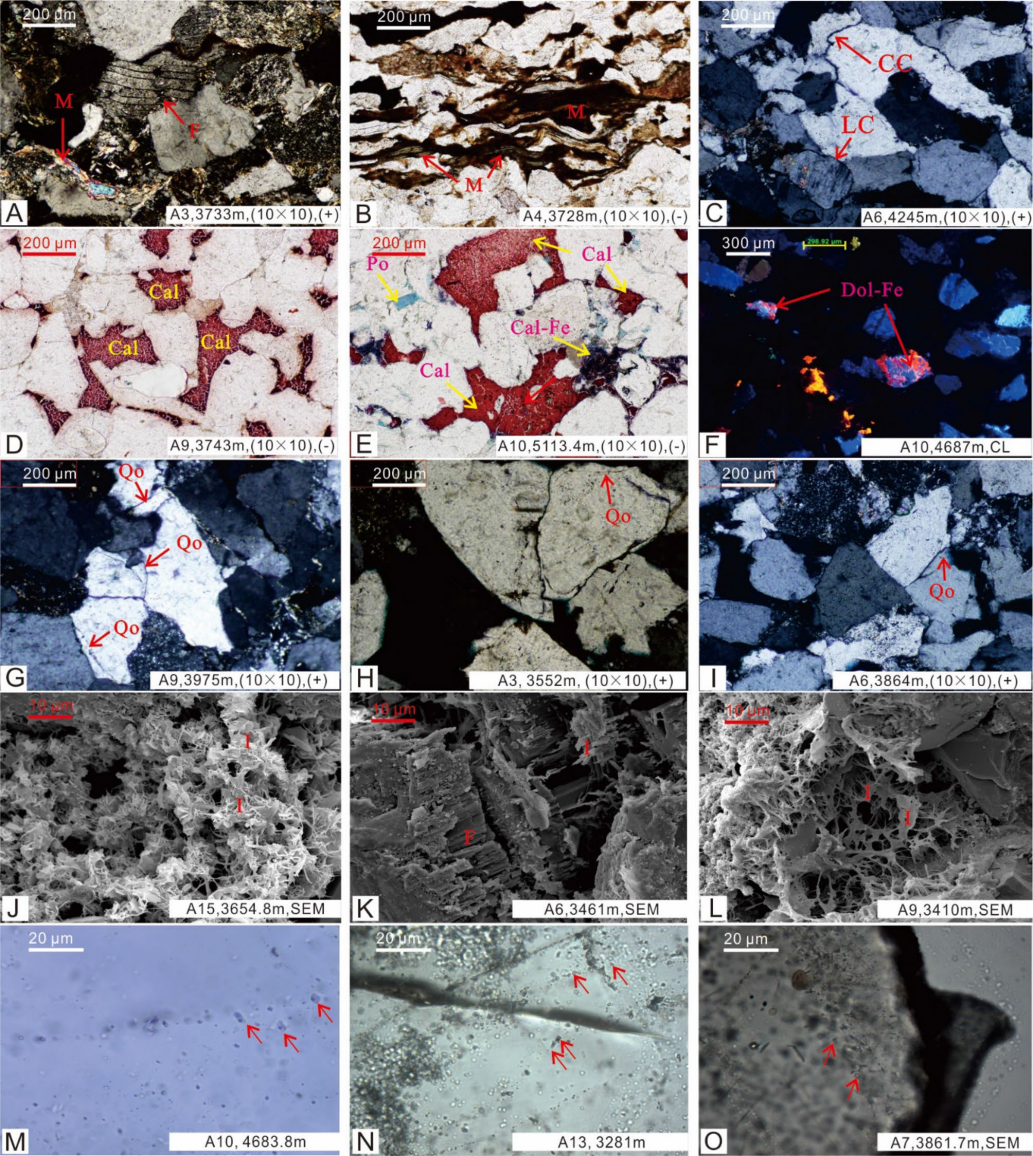
Microscopic characteristics of rock samples from Huagang Formation in Xihu sag. **(A–C)** Characteristics of reservoir compaction; **(D–F)** characteristics of authigenic carbonate minerals; **(G–I)** characteristics of authigenic illite minerals minerals; **(M–O)** inclusions on quartz transgranular crack and quartz overgrowth; *M *muscovite; *F *feldspar; *Qo* quartz overgrowth; *I *illite; *LC *line contact; *CC *concavo–convex contact.

The homogenization temperature (Th) of inclusions captured in quartz grains and secondary enlarged edge (Fig. 5M–O) can represent the paleotemperature of the reservoir, which can be well applied to the division of diagenetic stage^22^. The homogenization temperature analysis of the reservoir inclusions in the Western sag and Central uplift belt showed that most inclusions were formed in the stage IIA, and the maximum homogenization temperature is not more than 175 ℃ (Fig. 3B) (Table 2), indicating that some reservoirs have reached the the stage IIB.

### Maximum pyrolysis temperature Tmax

The maximum pyrolysis temperature of mudstone in Xihu sag is ranging from 402 to 479 ℃ (Fig. 3E) (Table 2). According to the classification standard of diagenetic stage of clastic rock, it can be concluded that the reservoir at present day is mainly in stage IIA–IIB.

## Discussion

### Comprehensive analysis of diagenetic stage

It can be seen under the microscope that the plastic components (biotite, muscovite, feldspar) have been deformed and oriented by strong mechanical compaction (Fig. 5A,B). In addition, the contact relationships between the detrital particles (line contacts, line-to-concavity contacts, concavity-convexity contacts, and suture line contacts) (Fig. 4C) also indicate that the Huagang formation is in a relatively high diagentic stage. The carbonate cementation mainly involved the calcite, dolomite, ferro calcite, ferrodolomite (Fig. 4D–F), and ankerite is a sign of late diagenetic stage^16^.

In consideration of formation temperature (Fig. 3A), temperature measurement of quartz mineral inclusions (Fig. 3B), vitrinite reflectance (*Ro*%) (Fig. 3C), clay mineral X-ray diffraction (XRD) (Fig. 3D), organic matter pyrolysis peak temperature (Tmax) (Fig. 3E), as well as the evolution characteristics of authigenic minerals, pore structure, grains contact relation observed under the microscope(Fig. 5), the reservoir diagenetic stage was identified. According to the classification standard of diagenetic stage of clastic rock in China's oil and gas industry, the reservoir of Huagang Formation in Xihu sag is mainly in stage IIA–IIB. It was showed that 2300–3200 m is in the stage IIA1, while 3200–4300 m is in the stage IIA2 and 4300–5000 m is in the stage IIB (Fig. 3).

### Diagenetic evolution of Huagang Formation

#### Analysis of burial history and thermal history

##### Analysis of burial history

**Figure 6:**
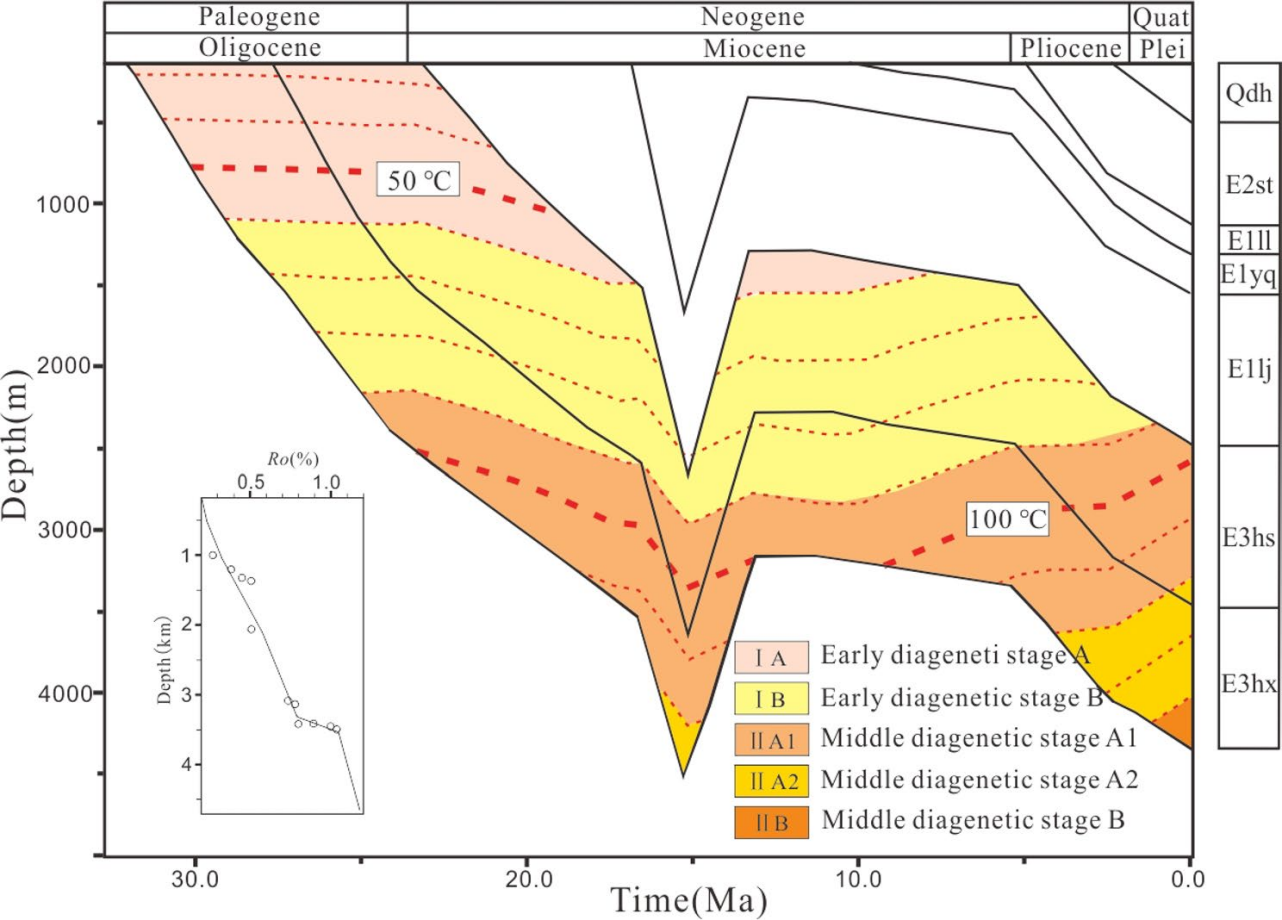
Stripping column profile of Well A9 in Xihu sag.

In the process of exploration and development of oil and gas fields, a lot of great efforts need to be made to analyze the formation and development of sedimentary strata and structures^22,41,42^. The total burial characteristic of the Oligocene Huagang Formation in Xihu sag is as follows: slow subsidence period (N1lj-N1yq) with burial rate raging from 92.4 to 202.2 m/Ma (av. 139.2 m/Ma), rapid subsidence period (N1yq-N1lyq) with burial rate raging from 297.9–501.3 m/Ma (av. 386.8 m/Ma), structural uplift period (N1yq-N1ll) with uplift rate raging form 123.7–281.8 m/Ma (av. 176.5 m/Ma) and stable subsidence period (N1ll-Qdhq) with burial rate raging form 39.5–84.5 m/Ma (av. 61.6 m/Ma) (Fig. 6) (Table 3). In BP1 stage, the CUB I has the fastest burial rate with 163.5–202.2 m/Ma, followed by CUB II with 145.8 m/Ma and WS with 114.3–174.1 m/Ma, and WSB and CUB III have the slowest burial rate, with 119.1–142.8 m/Ma and 55.3–165.1 m/Ma respectively; In BP2 stage, WS burial rate is 332.4–501.3 m/Ma, followed by CUB I with 300.8–442.3 m/Ma, and WSB with 329.7 to − 472.8 m/Ma, and CUB II and CUB III have the slowest burial rate, with 356.9 m/Ma and 297.9–356.9 m/Ma respectively; In BP3 stage, CUB uplift rate is − 202.3 to − 281.8 m/Ma, followed by CUB III with − 170.5 to − 178.3 m/Ma. In BP4 stage, the Huagang Formation subsides slowly with burial rate raging form 39.5–84.5 m/Ma. The subsidence center shows a trend of from CUB I to WS at shallow burial depth (Table 3).

**Table 3:** The Burial rate of Huagang Formation of Xihu sag in different burial periods.

Burial rate (m/Ma)	WSB				WS				CUB I			CUB II	CUB III	
	A1	A5	A8	A12	A3	A10	A11	A13	A6	A7	A9	A4	A2	A14
BP1	92.8	131.3	98.6	92.4	141.5	174.1	154.5	114.3	193.2	202.2	163.5	145.8	117.6	110.6
BP2	346.6	472.8	329.7	355.6	339.5	501.3	432.6	332.4	300.8	442.3	437.1	356.9	443.8	297.9
BP3	− 144.2	− 123.7	− 125.9	− 183.7	− 130.3	− 151.3	− 200.2	− 177.7	− 202.3	− 281.8	− 210.8	− 137.3	− 170.5	− 178.3
BP3	74.8	83.0	53.2	55.9	60.5	84.5	53.6	58.4	61.2	39.5	59.8	64.6	54.6	58.3

BP1-N1lj-N1yq; BP2-N1yq-N1lyq; BP3-N1yq -N1ll; BP4-N1ll-Qdhq; “−”represents the uplift of strata.

##### Analysis of thermal history

Previous researchers^43–45^ have done a lot of research on the thermal flow value of the East China Sea Shelf Basin. It was showed that the current thermal flow value of this area is between 50–87 mW/m^2^, with an average of 66.5 mW/m^2^, which is a normal thermal flow value area (Tong et al., 2009; Luan, 2003). Based on the reflectance and fission track data of the vitrinite, Yang (2004)^45^ used the paleogeothermal gradient method to recover the paleothermal flow in the East China Sea Shelf Basin at the end of Mesozoic, Paleocene, Oligocene and Miocene. The results showed that the current thermal flow in the Xihu sag is between 55.3 and 84.3 mW/m^2^, with an average value of 71.7 mW/m^2^. The highest paleothermal flow occurred at the end of Oligocene, ranging from 63.8 to 98.6 mW/m^2^, with an average of 83.4 mW/m^2^. It is a continuous heat flow reduction process from Oligocene to present.

**Figure 7:**
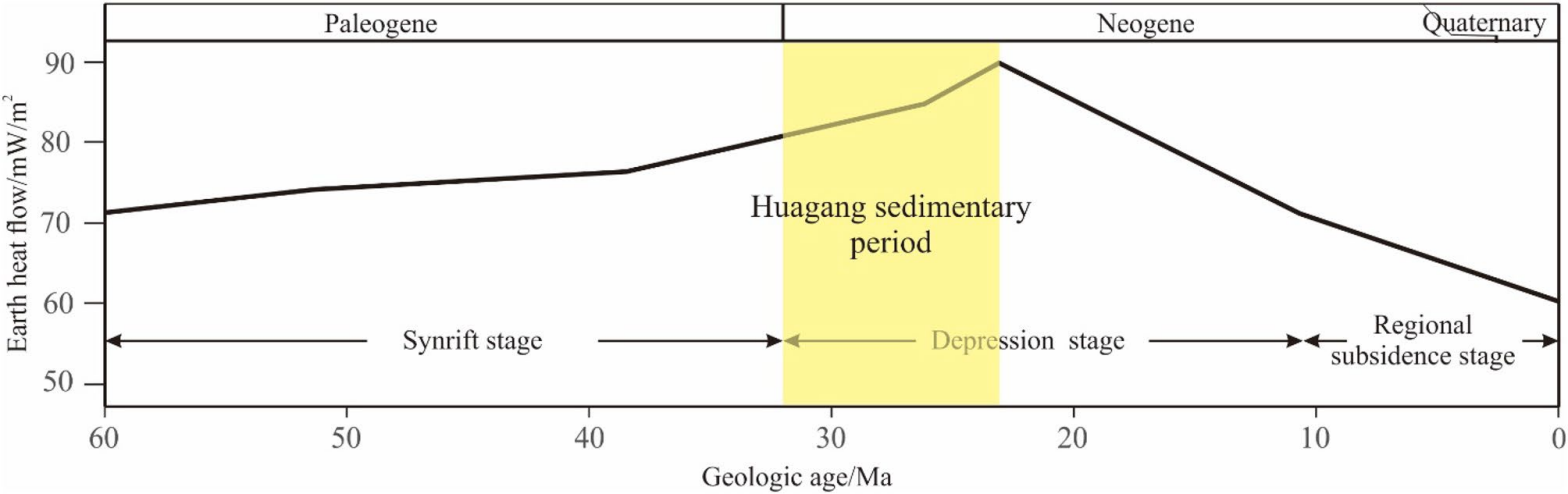
Geothermal flow value of Xihu sag in burial history.

Based on the above understanding, the general model of terrestrial heat flow in the thermal history simulation of Xihu sag is shown in Fig. 7. It can be seen from the figure that the deposition period of Huagang Formation belongs to the early stage of the compression sag stage, with the geothermal flow of 80.5 mW/m^2^, which gradually increased later. In the rifting stage, when the Longjing formation (N1L) was deposited, the geothermal flow reached the maximum of 90.0 mW/m^2^. Then, the geothermal flow began to decline gradually. During the deposition period of Liulang formation (N1ll), the geothermal flow decreased to 71.2 mW/m^2^. In the regional subsidence stage, the fault activity is weakened, and the heat flow of the earth is further reduced, which is maintained at about 60.0 mW/m^2^ (Fig. 7).

**Figure 8:**
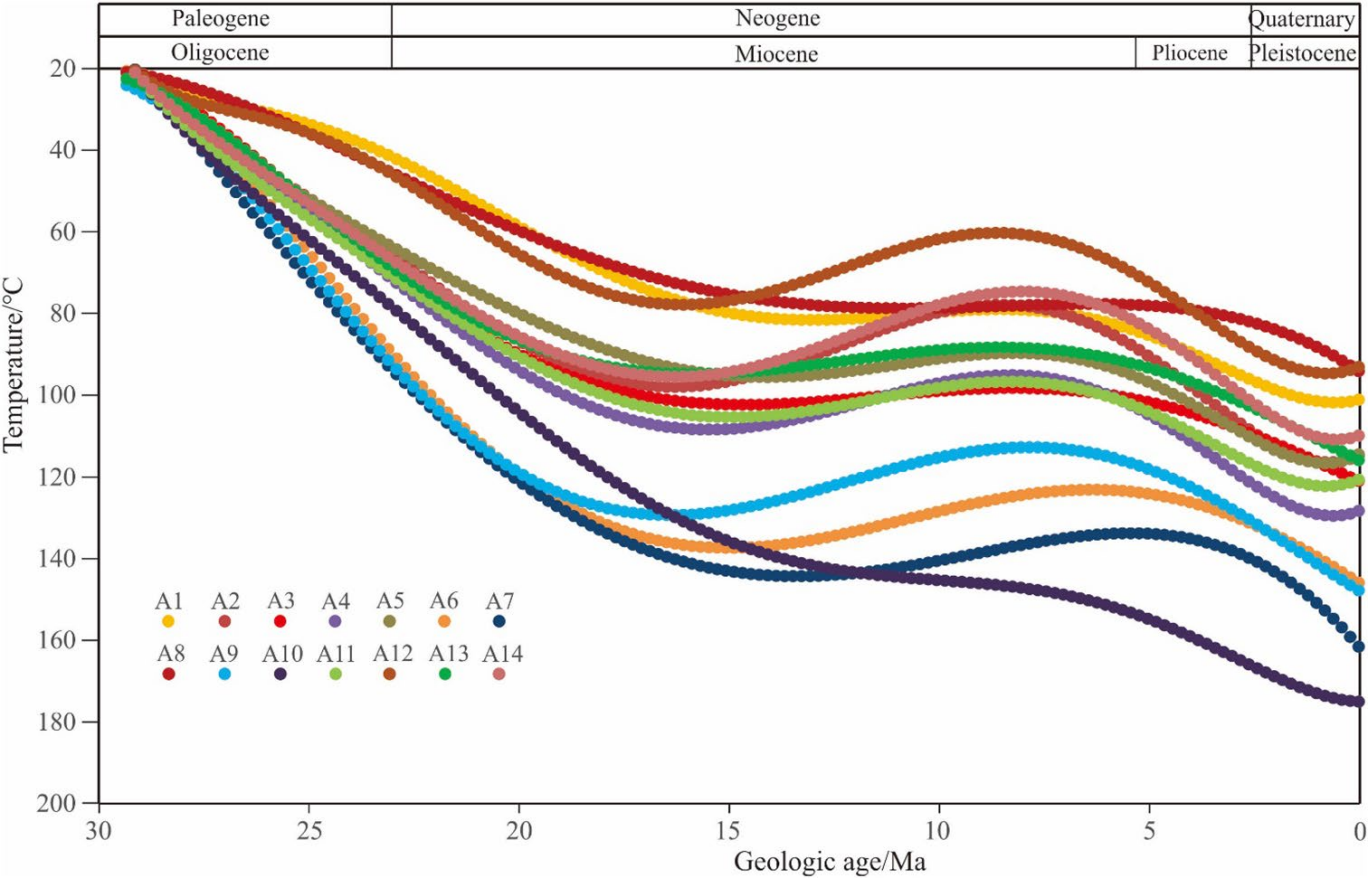
Paleo temperature of the top boundary of the seventh member of Huagang Formation in different structural blocks of Xihu sag.

The paleotemperature in the study area is generally distributed between 85 and 180 ℃, which is manifested in the rapid thermal evolution in the CUB I. During the deposition period of Longjing formation (N1lj), the formation temperature has exceeded 120 ℃, which is in stage IIA2, and then the temperature changes slowly with temperature variation rate less than 3 ℃/Ma. Until the deposition of the upper Pleistocene Santan formation (N2st), the temperature changes rapidly with temperature variation rate more than 5 ℃/Ma, and the formation rapidly evolves to the middle diagenetic stage IIB. In the CUB II and WS, the formation temperature has exceeded 85 ℃ in the deposition period of Longjing formation (N1ll), which is in stage IIA1. Then the temperature changes slowly all the time with temperature variation rate less than 2 ℃/Ma. By the deposition of the Santan formation (N2st) of the Pliocene, the temperature changes rapidly with temperature variation rate about 2 ℃/Ma, and the formation rapidly develops to the stage IIA2. The area with the lowest degree of thermal evolution is the west slope zone, and the simulated current temperature is generally low, ranging from 85 to 110 ℃ (Fig. 8).

##### Coupling relationship between thermal history and structural evolution

The rifting process of Xihu sag lasted from paleocene to the end of oligocene, and then transformed into thermal subsidence in Miocene, and a new round of rapid subsidence started again in Pliocene Pleistocene^23,33^. The time of rapid tectonic subsidence in Xihu sag corresponds to the highest paleoheat flow and the rapid rise of paleogeotemperature, which reflects the control effect of tectonic activity on the geothermal state of the basin.

#### Diagenetic parameter simulation

##### Transformation characteristic of clay minerals

**Figure 9:**
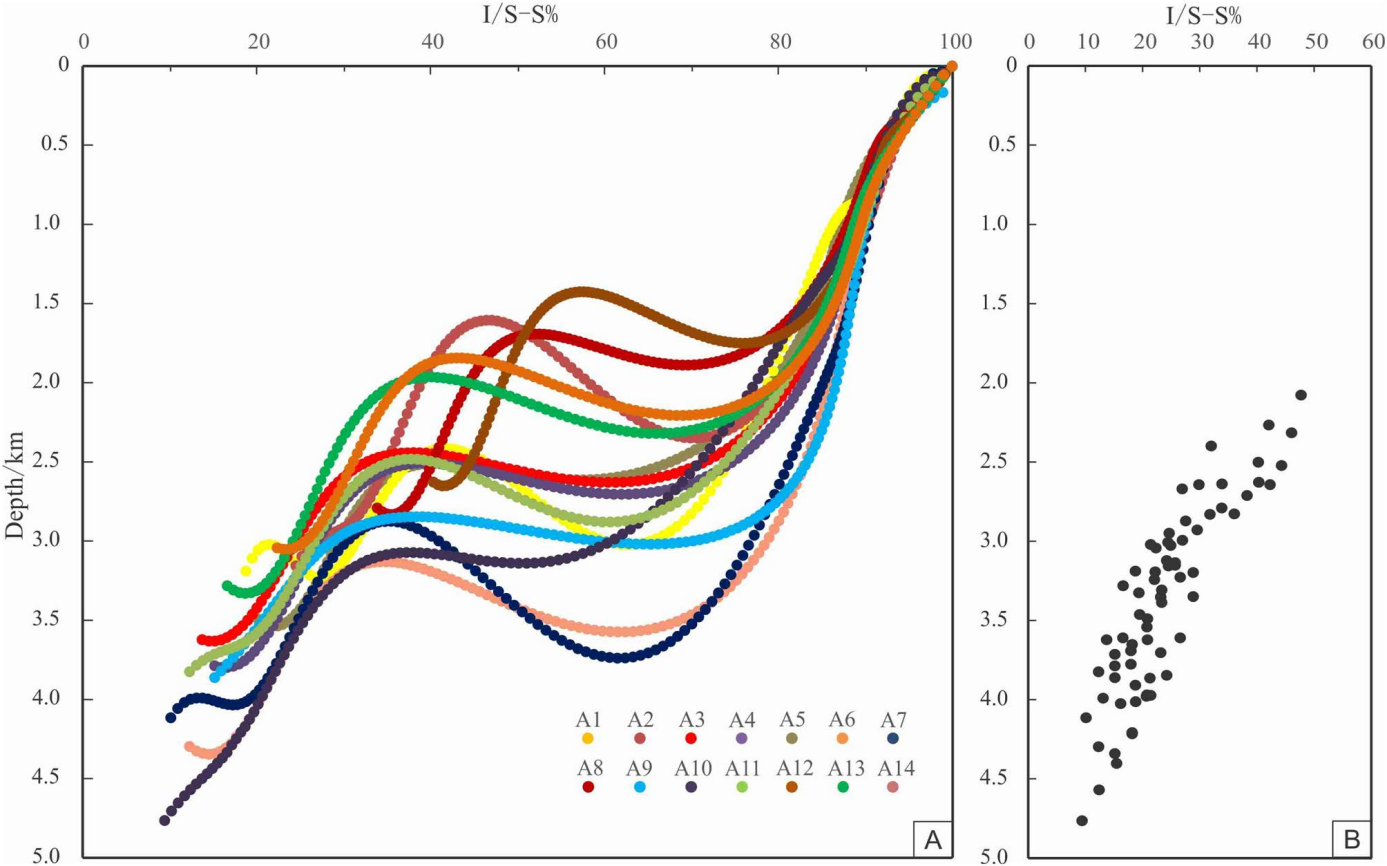
Thermal simulation of clay mineral transformation of Huagang Formation in Xihu sag. **(A)** I/S-S% in geologic time, **(B)** I/S-S% at present time based on simulation.

I/S mixed layer are widely distributed in mudstone and sandstone. The proportion of I/S-S% is an important means to judge the diagenetic strength and temperature of clastic rocks^21,38,39^. The transformation process from montmorillonite to illite mainly depends on time, temperature and pressure. With the increase of time, temperature and pressure, the transition sequence from montmorillonite to disordered I/S to ordered I/S and illite content increases gradually. The proportion I/S-S% decreased during burial diagenesis and geothermal alteration. The simulated value of I/S-S% in the study area ranges from 9.4 to 47.7%, with an average of 24.2%, which is in good agreement with the actual measured value in the study area. The value of I/S-S% indicates that the Huagang Formation in Xihu sag is now in stage IIA-IIB. In general, the I/S-S% value shows the following characteristics: (1) it changes slowly with a variation rate less than 2.5%/Ma in the early stage of burial and decreases rapidly with a variation rate about 15%/Ma when the depth exceeds 1500 m. The CUB I has the largest change range about 70%, followed by the CUB II with about 60% and WS with about 55%, while the Western slope has a relatively small change range with about 35%. In the later stage of burial, the change range of simulation value decreases again; (2) the older the strata in the buried history, the lower the I/S-S% value, and the I/S-S% of the CUB is lower than other areas with a value about15%, indicating relatively strong diagenesis in the CUB; (3) the uplift of the stratum does not affect the transformation of montmorillonite, but the change range is reduced. Previous studies^20,21^ have shown that the transformation rate of montmorillonite to illite is closely related to the geothermal gradient. When the temperature is high, shallow burial can lead to rapid transformation, and the converse is also true. In addition, the transformation from montmorillonite to illite is also very fast in the water medium of the reservoir under the condition of rich potassium. Among various influencing factors, strong basic thermal activity and high geothermal gradient are the main reasons for low I/S-S% (Fig. 9).

##### Evolution characteristics of vitrinite reflectance

**Figure 10:**
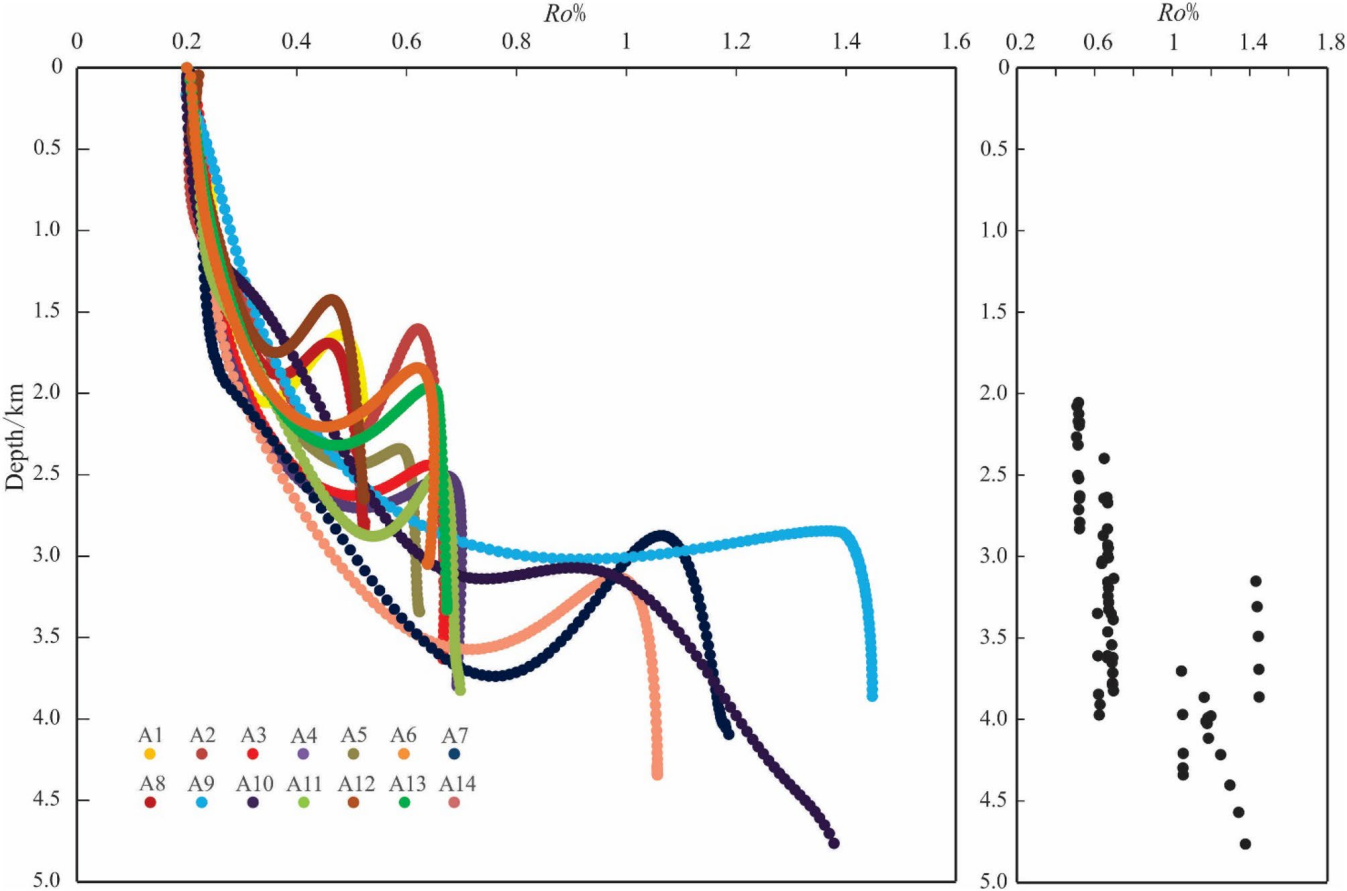
Thermal simulation of organic matter maturity of Huagang Formation in Xihu sag. **(A)** *Ro*% in geologic time, **(B)** *Ro*%at present time based on simulation.

The simulated reflectance (*Ro*%) of vitrinite ranges from 0.51 to 1.45%, with an average of 0.81% (Fig. 10). *Ro*% indicates that the reservoir of Huagang Formation in the study area is in IIA-IIB. In general, *Ro*% value shows the following characteristics: (1) it changes slowly with a variation rate about 0.025%/Ma in the early stage of burial, and the simulation value increased rapidly with a variation rate about 0.075%/Ma when the depth is more than 2000 m. The CUB I has the largest change range, followed by CUB II and WS, and the WSB has a relatively small change range with a variation rate about 0.05%/Ma. In the later stage of burial, the change range of simulation value decreases again. (2) the older the strata in the buried history, the higher the *Ro*%, and the *Ro*% of the Central uplift belt in the study area is the largest with a value about 1.2%. The results show that the maturity of organic matter is high and diagenesis is relatively strong; (3) the uplift of strata does not affect the thermal evolution of organic matter, and the evolution of vitrinite formation is irreversible. Vitrinite reflectance is mainly controlled by temperature, and also affected by diagenetic time, tectonic activity and magmatic activity^17–37^^.^

#### The fine subsection of diagenetic stage (DSF)

In the past decades, many scholars at home and abroad^6,10,17,18,46^ have made extensive and in-depth research on the diagenesis of clastic rocks, and put forward corresponding diagenetic stage division standard of clastic reservoir. In the early 1990s, on the base of the domestic and foreign scholars^6,10,47^ on diagenesis research and diagenetic stage division, Ying Fengxiang (1992)^48^ combined with the specific geological characteristics of sedimentary basins in China systematically and brought forward the specification for diagenetic stage division of clastic rocks. In this division scheme, the diagenetic stage of clastic rocks was based on the thermal evolution model of organic matter, distribution characteristics of clay minerals and evolution law of sandstone authigenic minerals and divided into early diagenetic stage and late diagenetic stage. Another scheme from Wilson M. D. (1994)^10^ was based on the study of clastic rock diagenesis of many scholars in the world and on account of organic matter thermal evolution, which divided the organic matter thermal evolution into immature stage, decarboxylation stage, liquid hydrocarbon generation stage and the natural gas generation stage to match the corresponding diagenetic temperature and autogenetic mineral evolution sequence for different organic matter thermal evolution stages. At the beginning of the twenty-first century, Ying revised the classification standard of the early diagenetic stage of the clastic rock and divided the diagenetic evolution of the clastic rock into three stages^19^. In this new scheme, the intermediate diagenetic stage was subdivided into two stages, A and B, and the intermediate diagenetic stage A was further divided into two sub stages, A1 and A2 (Table 1). This division standard of diagenetic stage of clastic rock has been used up to now.

The accurate division of diagenetic stages brings insight into the diffidence of paleo temperature (T), vitrinite reflectance (*Ro*%), the highest pyrolysis peak temperature (Tmax), thermal alteration index of the proportion of smectite in illite/smectite interstratified minerals (I/S-S%) and authigenic minerals assemblage in both time domain and space domain^11,24,49^. In order to accurately describe the diagenetic evolution process of Huagang Formation, a fine division scheme of diagenetic stage of clastic reservoir (Table 4) with 36 diagenetic micro-stage (DS) in diageneitic process was proposed based on the paleotemperature, vitrinite reflectance and the proportion of smectite in illite/smectite interstratified minerals. DS1–DS9 is the equal of IA, while DS10–DS13, DS14–DS19, DS20–DS24, DS25–DS31 and DS32–DS36 are equivalent to the stage IB, IIA1, IIA2, IIB and III separately. This fine division scheme can be used for numerical simulation of reservoir diagenetic evolution, which can highlight the differences of reservoir evolution process and help to identify the key geological factors linked to the differential evolution of high diageneisis reservoir.

**Table 4:** A fine division scheme of diagenetic stage of clastic reservoir.

DS	Temperature (℃)	*Ro*%	I/S-S%	DS	Temperature (℃)	*Ro*%	I/S-S%
DS1	≤ 25	< 0.24	> 99–100	DS19	> 110–115	< 0.95–0.87	> 27–31
DS2	> 25–30	< 0.26–0.24	> 96–99	DS20	> 115–120	< 1.03–0.95	> 24–27
DS3	> 30–35	< 0.28–0.26	> 94–96	DS21	> 120–125	< 1.11–1.03	> 21–24
DS4	> 35–40	< 0.30–0.28	> 90–94	DS22	> 125–130	< 1.18–1.11	> 19–21
DS5	> 40–45	< 0.34–0.30	> 87–90	DS23	> 130–135	< 1.25–1.18	> 16–19
DS6	> 45–50	< 0.35–0.34	> 83–87	DS24	> 135–140	< 1.31–1.25	> 14–16
DS7	> 50–55	< 0.36–0.35	> 78–83	DS25	> 140–145	< 1.38–1.31	> 13–14
DS8	> 55–60	< 0.37–0.36	> 74–78	DS26	> 145–150	< 1.45–1.38	> 11–13
DS9	> 60–65	< 0.39–0.37	> 69–74	DS27	> 150–155	< 1.52–1.45	> 10–11
DS10	> 65–70	< 0.40–0.39	> 65–69	DS28	> 155–160	< 1.61–1.52	> 9–10
DS11	> 70–75	< 0.42–0.40	> 60–65	DS29	> 160–165	< 1.72–1.61	> 8–9
DS12	> 75–80	< 0.45–0.42	> 55–60	DS30	> 165–170	< 1.86–1.72	> 7–8
DS13	> 80–85	< 0.50–0.45	> 51–55	DS31	> 170–175	< 2.04–1.86	> 6–7
DS14	> 85–90	< 0.56–0.50	> 46–51	DS32	> 175–180	< 2.27–2.04	> 5–6
DS15	> 90–95	< 0.64–0.56	> 42–46	DS33	> 180–185	< 2.57–2.27	> 4–5
DS16	> 95–100	< 0.71–0.64	> 38–42	DS34	> 185–190	< 2.96–2.57	> 3–4
DS17	> 100–105	< 0.79–0.71	> 34–38	DS35	> 190–195	< 3.45–2.96	> 1–3
DS18	> 105–110	< 0.87–0.79	> 31–34	DS36	> 195–200	4.00–3.45	0–1

**Figure 11:**
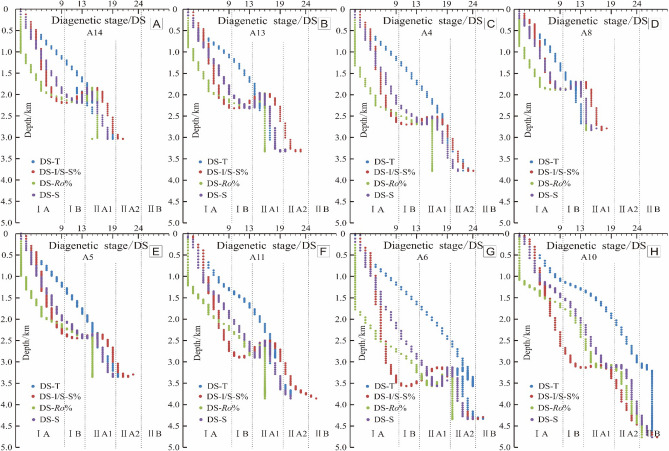
Comparative analysis of diagenetic stages using different diagenetic parameter.

**Figure 12:**
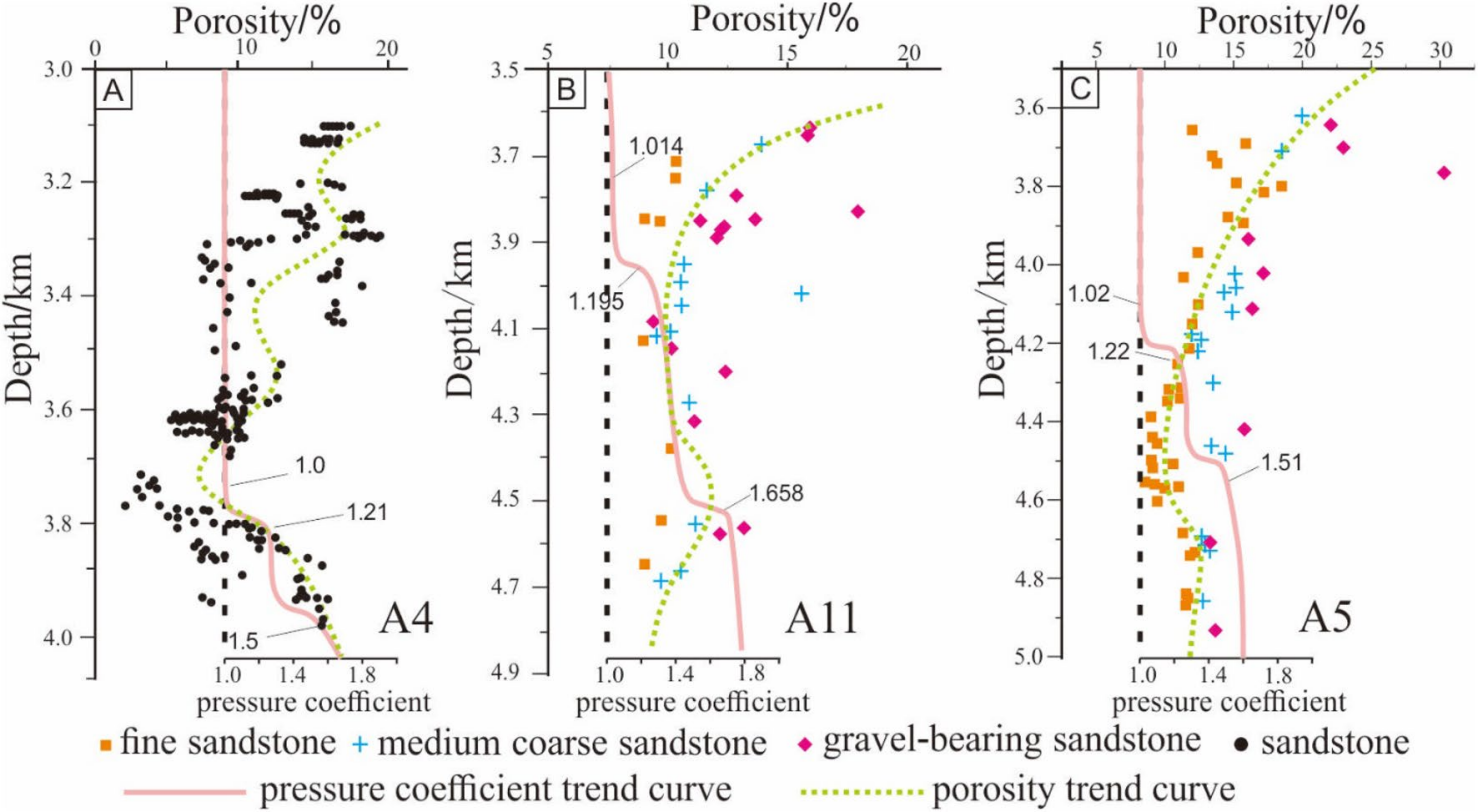
Relationship between porosity and depth in abnormal high pressure wells in Xihu sag.

The simulation results of Paleotemperature (T), vitrinite reflectance (*Ro*%) and I/S–S% show that the reservoir of Huagang Formation in the study area is mainly in stage IIA1, and stage IIA2, and some reservoirs in the north Central uplift belt and Western sag such as A10 well area have entered stage IIB (Fig. 11). The diagenetic stages indicated by different diagenetic parameters have the following characteristics: (1) in the shallow burial phase (H < 1000 m, T < 60 ℃), the diagenetic stages divided by paleotemperature (DS-T) and smectite in mixed layer (DS-I/S-S%) are equivalent or close and they are higher than that of diagenetic stages divided by vitrinite reflectance (DS-*Ro*%); (2) as the depth of stratum increased (H < 2000 m, T < 100 ℃), the difference between DS-T and DS-I/S-S% increased at the same time, which is shown as that DS-T is significantly greater than DS-I/S-S% and DS-*Ro*%, and DS-*Ro*% is minimum; (3) when the burial depth is further increased, DS-I/S-S%, DS-*Ro*% and DS-T tend to have the same value DS. Most of the well areas appear in stage IIA1, and some well areas such as A8 appear in stage IIB. The depth and location of DS are different in different structural areas; (4) after the poit DS, with the increase of burial depth, DS-*Ro*% no longer changes, or changes very little. However, DS-I/S-S% varies greatly with the increase of burial depth, and DS-I/S-S% is significantly larger than DS-T and DS-*Ro*%, and DS-*Ro*% is minimum. However, exceptional conditions occured in well A10. After DS stage, DS-I/S-S% changed little with the increase of burial depth, and DS-T was still the largest, followed by DS-*Ro*%, and the lowest is DS-I/S-S%. According to the measured data from the oil field (Fig. 12), there are abnormal overpressure environments in the well A4, well A11 and well A5. The initial depth values of abnormal overpressure are 3800 m, 3900 m and 4200 m respectively, and with the increase of depth, the formation pressure coefficients are measured to be up to 1.8. Previous studies^50,51^ on the evolution of organic matter and the transformation of clay minerals in overpressure environment have shown that the increase of pressure can inhibit the evolution of organic matter and hydrocarbon generation, and reduce the transformation of montmorillonite to illite^52,53^. The abnormal overpressure may begin to develop at the burial depth of 3000 in well A10. The existence of the abnormal overpressure restrains the transformation of montmorillonite to illite, leading to the DS-I/S-S% value relatively smaller after DS; (5) in the diagenetic process, with the increase of temperature, the transformation of montmorillonite to illite is more sensitive than the evolution of organic matter, and DS-I/S-S% can effectively reflect the diagenetic process. In the shallow burial stage, the description of diagenetic evolution stage is relatively accurate compared with vitrinite and paleotemperature, while in the deep burial process, due to the existence of abnormal overpressure, DS-I/S-S% is relatively small.

It is generally believed that time and temperature are the main reaction processes in the evolution of diagenesis. Therefore, the rapid deposition and burial of strata can slow down diagenesis. Diagenesis is not synchronous with temperature. The current diagenetic stage is a comprehensive product of the whole geological history. When different indicators are used to judge the diagenetic stage, the prediction results are different in time and space (Fig. 11). Because the depth, temperature, duration, pressure and some chemical variables can change synchronously or asynchronously, it is difficult to determine the parameters that affect the evolution series of apparent diagenesis stage. Therefore, in order to accurately describe diagenesis and achieve the goal of successful oil and gas exploration, a variety of comprehensive prediction indexes should be needed.

## Conclusions

(1) Based on the comprehensive utilization of formation temperature data, inclusion temperature data, vitrinite reflectance test data, organic thermal solution peak temperature data and clay mineral X-ray diffraction data, combined with the analysis of rock physical properties, particle contact mode, pore structure, pore type and other characteristics, the diagenetic evolution stage of Huagang Formation reservoir was analyzed. It was showed that the reservoir of Huagang Formation in Xihu sag is mainly in stage IIA-IIB. Among them, 2300–3200 m is in stage IIA1, 3200–4300 m is in stage IIA2 and 4300–5000 m is in stage IIB.

(2) The burial history and thermal evolution history of the study area are restored. On this basis, paleogeotemperature, vitrinite reflectance and smectite in illite/smectite interstratified minerals were selected to simulate the evolution of Huagang clastic rocks. The simulation results showed that most of the strata in the upper part of Huagang Formation (HS) of Xihu sag are in the stage IIA1-IIA2. The CUB I is deeply buried, and some of the strata have entered the stage IIB. The lower part of Huagang Formation (HX) in Xihu sag is in the stage IIA1–IIB, in which the WSB is in the stage IIA1, while the WS and CUB III are mainly in the stage IIA2, and the CUB I and CUB II is in the stage IIB.

(3) A fine subsection scheme of 36 diagenetic micro-stage (DS) in diageneitic process was proposed based on the paleotemperature, vitrinite reflectance and the proportion of smectite in illite/smectite interstratified minerals. DS1–DS9 is the equal of IA, while DS10–DS13, DS14–DS19, DS20–DS24, DS25–DS31 and DS32–DS36 are equivalent to the stage IB, IIA1, IIA2, IIB and III separately. This fine division scheme can be used for numerical simulation of reservoir diagenetic evolution, which can highlight the differences of reservoir evolution process and help to identify the key geological factors linked to the differential evolution of high diageneisis reservoir.

(4) When judging the diagenetic stage of reservoir in geological period based on a single index, the prediction results of different parameters are quite different. This difference reflects the influence of structure and abnormal pressure on diagenetic evolution. In order to accurately describe the diagenetic evolution process of Huagang Formation, three parameters of T, *Ro*% and I/S-S% were used for a fine discrimination model of diagenetic stage.
